# Spousal associations of serum metabolomic profiles by nuclear magnetic resonance spectroscopy

**DOI:** 10.1038/s41598-021-00531-z

**Published:** 2021-11-03

**Authors:** Karema Al Rashid, Neil Goulding, Amy Taylor, Mary Ann Lumsden, Deborah A. Lawlor, Scott M. Nelson

**Affiliations:** 1 School of Medicine, University of Glasgow, New Lister Building, Glasgow Royal Infirmary, Glasgow, G31 2ER UK; 2 MRC Integrative Epidemiology Unit at the University of Bristol, Bristol, BS8 2BN UK; 3 Bristol Medical School, Population Health Science, Bristol, UK; 4 NIHR Bristol Biomedical Research Centre, Bristol, UK

**Keywords:** Endocrine system and metabolic diseases, Epidemiology

## Abstract

Phenotype-based assortative mating is well established in humans, with the potential for further convergence through a shared environment. To assess the correlation within infertile couples of physical, social, and behavioural characteristics and 155 circulating metabolic measures. Cross sectional study at a tertiary medical center of 326 couples undertaking IVF. Serum lipids, lipoprotein subclasses, and low-molecular weight metabolites as quantified by NMR spectroscopy (155 metabolic measures). Multivariable and quantile regression correlations within couples of metabolite profiles. Couples exhibited statistical correlations of varying strength for most physical, social, and behavioural characteristics including age, height, alcohol consumption, education, smoking status, physical activity, family history and ethnicity, with correlation coefficients ranging from 0.22 to 0.73. There was no evidence of within couple associations for BMI and weight, where the correlation coefficients were − 0.03 (95% CI − 0.14, 0.08) and 0.01 (95% CI − 0.10, 0.12), respectively*.* Within spousal associations of the metabolite measurements were all positive but with weak to modest magnitudes, with the median correlation coefficient across all 155 measures being 0.12 (range 0.01–0.37 and interquartile range 0.10–0.18). With just four having associations stronger than 0.3: docosahexaenoic acid (0.37, 95% CI 0.22, 0.52), omega-3 fatty acids (0.32, 95% CI 0.20, 0.43) histidine (0.32, 95% CI 0.23, 0.41) and pyruvate (0.32, 95% CI 0.22, 0.43). Infertile couples exhibit spousal similarities for a range of demographic and serum metabolite measures, supporting initial assortative mating, with diet-derived metabolites suggesting possible subsequent convergence of their individual metabolic profile.

## Introduction

Phenotype-based assortative mating is well established in humans for several traits including age^1,2^, height^3,4^ and other physical characteristics such as skin pigmentation^5^, eye and hair colour^6^. In addition, there are other behavioural and social factors that are correlated between spouse-pairs and are thought to affect mate selection such as educational level^2,7^, occupation^2^, socio-economic status^1^, smoking^8^, alcohol consumption^9^, language and culture^10^. For other physical and physiological characteristics such as weight^7^, body mass index^4^ and blood pressure^8^, weak to modest positive correlations are also observed, potentially reflecting both initial assortative mating, and subsequent spousal interaction and convergence through a shared environment and behaviours^11^.

Over the last decade epidemiological studies have increasingly measured circulating multiple metabolic traits, which collectively provide information on genomic, environmental and lifestyle traits. As physical, social and behavioural assortative mating traits may be associated with these metabolic profiles, correlations between spouses for a range of metabolites may be anticipated. However, despite the long established positive correlations of physical, social, and behavioural characteristics between couples, the assessment of metabolic measures in couples has been limited and primarily focused on conventional cardiovascular risk factors^8^. For example, for total cholesterol, LDL cholesterol, and triglycerides, the within-spouse correlation coefficients are generally weak with coefficients ranging from 0.05 to 0.10^8^, with limited evidence of correlation for glucose and HDL cholesterol^8^.

Serum nuclear magnetic resonance (NMR) metabolomics enables reproducible quantification of circulating lipids and abundant metabolites^12^ and has been used to assess the differences in metabolites with adiposity^13^, height^14^, glycemia^15^ and a range of physiological and pathogenic disease states^16–18^. Furthermore, detailed metabolic profiling has been applied to assess the heritability and genetic architecture of blood metabolites^19–22^, that may underlie established physical, social and behavioural assortative mating traits. We could only identify three studies assessing four cohorts for within-spouse metabolite correlations. These included sample sizes of 281, 327, 64 and 6 spousal pairs, respectively, used different metabolite platforms to each other and the one we use here, which covered 120, 110, 51 and 147 metabolic trait measures and were undertaken in extended families of twin studies^19–21^. In general, they found weak metabolic trait spousal correlations (mean = 0.08, 0.18, 0.24), with these being weaker than twin correlations.

The aim of the current study was to assess the correlation within couples undergoing fertility treatment of physical, social and behaviour characteristics, and 155 circulating metabolic measures. These measures were profiled by a high throughput cost efficient NMR platform, covering a range of metabolic pathways, predominantly a lipidome, including lipoprotein lipids, fatty acids, as well as some amino acids, ketone bodies, and glycaemic traits. Whilst other studies have explored spousal similarity of the physical, social and behavioural characteristics that we also explore here, it is important that we explore these in this group of couples to help interpretation of metabolite correlations. Specifically, if we see similar correlations in these infertile couples to those seen in general populations it provides some rationale for assuming the results for the metabolites might generalise to a more general population.

## Materials and methods

### Study design and participants

**Figure 1:**
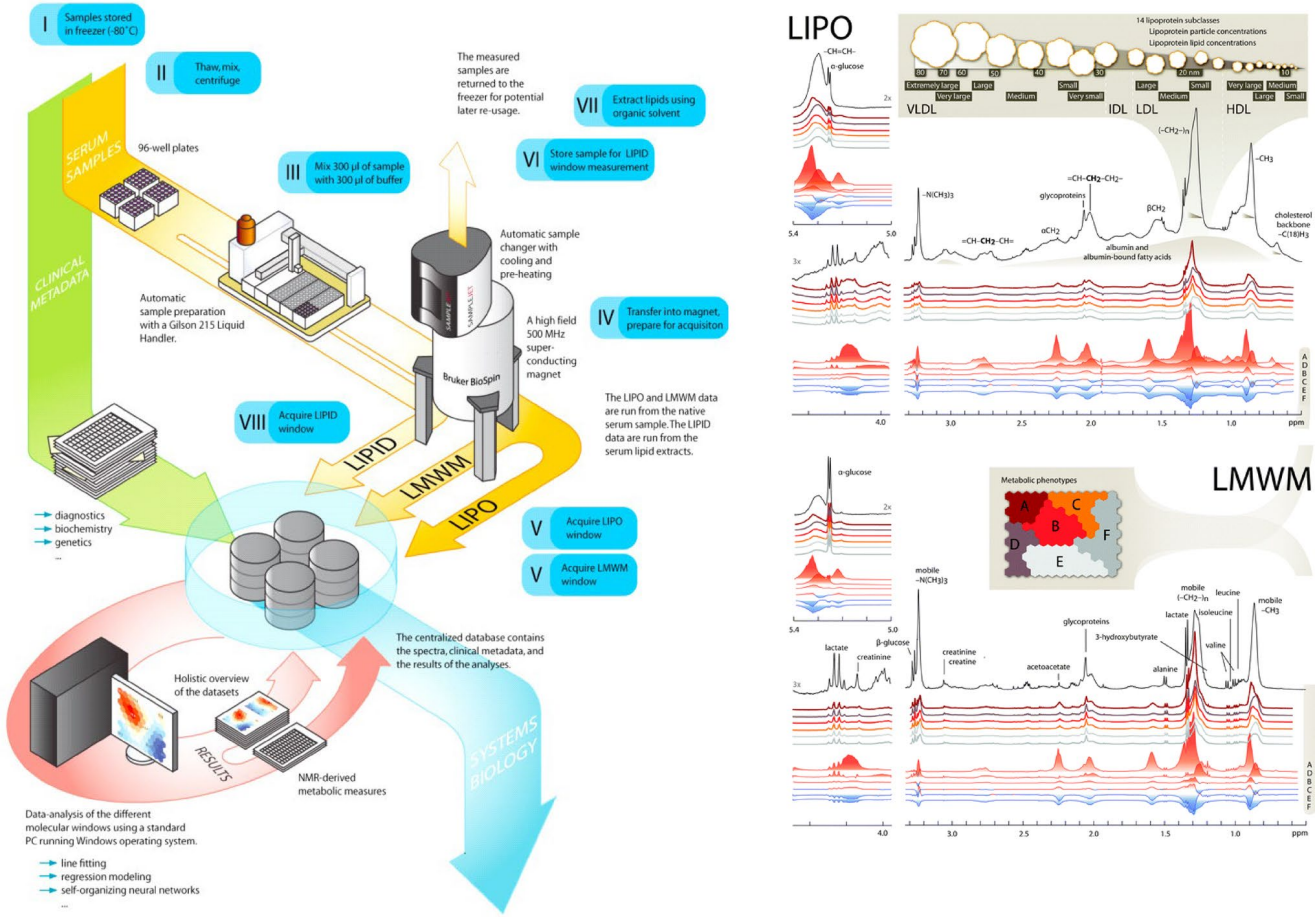
Summary of NMR spectroscopy method. The NMR spectroscopy methodology utilises approach uses three molecular windows, (two that were applied to native serum and one to serum lipid extracts requiring minimal preparation) to quantify the 148 metabolic traits. The NMR-based metabolite quantification is achieved through measurements of three molecular windows from each serum sample. Two of the spectra (LIPO and LMWM windows) are acquired from native serum and one spectrum from serum lipid extracts (LIPID window). The NMR spectra are measured using Bruker AVANCE III spectrometer operating at 500 or 600 MHz. Measurements of native serum samples and serum lipid extracts are conducted at 37 °C and 22 °C, respectively. The LIPO window represents a standard spectrum of human serum displaying broad overlapping resonances arising from lipid molecules in various lipoprotein particles. The LIPO data are recorded using 8 transients acquired using a NOESY-presat pulse sequence with mixing time of 10 ms and water peak suppression. The LMWM window includes signals from various low-molecular-weight molecules. The LMWM spectrum is recorded using a relaxation-filtered pulse sequence that suppresses most of the broad macromolecule and lipid signals to enhance detection of small solutes. Specifically, a Carr–Purcell–Meiboom–Gill (CPMG) pulse sequence with a 78 ms T2-filter and fixed echo delay of 403 μs is applied using 24 transients. The LIPID window of the serum extracts is acquired with a standard 1D spectrum using 32 transients. QC and outputs The NMR spectra were analysed for absolute metabolite quantification (molar concentration) in an automated fashion. For each metabolite a ridge regression model was applied for quantification in order to overcome the problems of heavily overlapping spectral data. In the case of the lipoprotein lipid data, quantification models were calibrated using high performance liquid chromatography methods, and individually cross-validated against NMR-independent lipid data. Low-molecular-weight metabolites, as well as lipid extract measures, were quantified as mmol/l based on regression modelling calibrated against a set of manually fitted metabolite measures. The calibration data are quantified based on iterative line-shape fitting analysis using PERCH NMR software (PERCH Solutions Ltd., Kuopio, Finland). Absolute quantification cannot be directly established for the lipid extract measures due to experimental variation in the lipid extraction protocol. Therefore, serum extract metabolites are scaled via the total cholesterol as quantified from the native serum LIPO spectrum. Figure adapted from^12^.

Cross-sectional study of women aged 18 to 45 and their male partners who presented at Glasgow Royal Infirmary, UK for assessment prior to assisted conception between 1 April 2017 and 31 March 2019^23,24^. Referral for state-funded assisted conception is limited to those where the female body mass index (BMI) ≤ 30 kg/m^2^ and both partners are non-smokers and in a stable relationship defined as cohabiting for ≥ 2 years, while for self-funding patients female BMI should be < 35 kg/m^2^. Exclusion criteria for study participation were a documented positive pregnancy test at time of presentation and /or requiring gamete or embryo donation. A total of 399 women were recruited, 326 of whom had a male partner who agreed to participate and of those 326 couples (100%) had a blood sample suitable for NMR analyses (Fig. 1).

The study was conducted according to ICH Guideline for good clinical practice, the Declaration of Helsinki and the Convention of the Council of Europe. All participants provided written informed consent. The study protocol was approved prior to study initiation by the NHS Health Research Authority (Ref 16/WM/0308).

### Study procedures

Physical, social, behavioural, fertility and medical history was obtained by self-reported questionnaire at the baseline visit or from the medical notes at initial recruitment^23,24^. Weight and height [used to calculate the body mass index (BMI)] were measured in light clothing and unshod. Weight was measured to the nearest 0.1 kg using Tanita scales; height was measured to the nearest 0.1 cm using a Harpenden stadiometer. Smoking status was categorised as ever versus never (to be considered for state funded assisted conception both women and men had to have not smoked for at least 3 months and this was confirmed by a negative cotinine breath test).

Non-fasted blood samples were collected during the same baseline visit for NMR analyses and immediately spun and frozen at − 80 °C and all NMR assays completed for this study were undertaken within 1 year of storage and with no previous freeze/thaw cycles.

### NMR protocol

Profiling of 155 lipid and metabolite measures was performed by a high-throughput targeted NMR platform [Nightingale Health© (Helsinki, Finland)] at the University of Bristol^23,24^. The platform applies a single experimental setup, which allows for the simultaneous quantification of routine lipids, 14 lipoprotein subclasses and individual lipids transported by these particles, multiple fatty acids, glucose, the glycolysis precursors lactate and pyruvate, ketone bodies, and amino acids in absolute concentration units (mostly mmol/l) (Fig. 1). The NMR-based metabolite quantification is achieved through measurements of three molecular windows from each sample. Two of the spectra (LIPO and LMWM windows) are acquired from native serum and one spectrum from serum lipid extracts (LIPID window). The NMR spectra were measured using Bruker AVANCE III spectrometer operating at 600 MHz. Measurements of native serum samples and serum lipid extracts are conducted at 37 °C and 22 °C, respectively. Details of this platform have been published previously^12,25^ and it has been widely applied in genetic and observational epidemiological studies^13,16–18,26–30^. Further details of the platform are provided in the Supplemental Material (Supplemental text and Table S1).

### Metabolite quantification and quality control

The NMR spectra were analysed for absolute metabolite quantification (molar concentration) in an automated fashion^23,24^. For each metabolite a ridge regression model was applied for quantification in order to overcome the problems of heavily overlapping spectral data. In the case of the lipoprotein lipid data, quantification models were calibrated using high performance liquid chromatography methods, and individually cross-validated against NMR-independent lipid data. Low-molecular-weight metabolites, as well as lipid extract measures, were quantified as mmol/l based on regression modelling calibrated against a set of manually fitted metabolite measures. The calibration data are quantified based on iterative line-shape fitting analysis using PERCH NMR software (PERCH Solutions Ltd., Kuopio, Finland). Absolute quantification cannot be directly established for the lipid extract measures due to experimental variation in the lipid extraction protocol. Therefore, serum extract metabolites are scaled via the total cholesterol as quantified from the native serum LIPO spectrum.

### Statistical analysis

All analyses were performed in Stata (version 15.1, StataCorp. 2017 College Station, TX), with figures created in R 4.0.2 (R Foundation for Statistical Computing, Vienna, Austria). Characteristics were summarized as n, total range, mean, standard deviation, median, and 25th and 75th quantiles (IQR) as appropriate. Associations between physical, social and behavioural characteristics within couples were investigated using both Pearson and Spearman’s rank correlation (continuous variables) and phi for dichotomized categorical variables^31^. In females and males separately, the metabolic measures were scaled to standard deviation (SD) units (by subtracting the mean and dividing by the standard deviation of all women or men respectively included in the analyses). This scaling allows easy comparison of multiple metabolic measures with different units or with large differences in their concentration distributions. Our primary analyses were the correlation of the unadjusted metabolite measures. In secondary analyses we assessed whether these were influenced by potentially similar physical, social and behavioural characteristics. This was done by using linear regression, with robust standard errors as some metabolite concentrations had skewed distributions, the sex-specific standard deviation (SD) scores of metabolites on a priori selected characteristics that might result in spousal correlations (age, educational attainment, ever smoking, physical activity, family history of cardiometabolic disease, alcohol consumption, BMI, ethnicity) and obtaining the residuals from these regression models (i.e. the sex specific metabolic concentrations having removed variation due to the observed characteristics listed above). The correlations between the residuals from these regressions within couples were then calculated using both Pearson and Spearman’s rank correlation. Confidence intervals for these correlations were calculated using bootstrapping. We used Student’s T-test to provide p values for the correlations. To test whether the skewness of the metabolite data was having any impact on correlations, regressions were repeated using quantile regression and results compared. To assess whether observed physical, social and behavioural characteristics that have been shown to correlate in couples explained any metabolite correlations we compared the covariates adjusted and unadjusted correlation using a scatter plot of these and exploring the linear fit. As women are excluded from infertility treatment if their BMI is greater than 30 kg/m^2^ and this exclusion (which is not applied to their male partners) may influence spousal correlations, we reassessed the correlation of spousal BMI after exclusion of the 17 male partners with a BMI > 30 kg/m^2^. Lastly, in additional analyses we ran principal component analyses in women and men separately and descriptively compared the number of principal components selected in women and men, the extent of overlap in factors loading on them and between spousal correlations for the top 10 components.

### Ethics approval and consent to participate

NHS Health Research Authority provided ethical approval for the study. REC reference 16/WM/0308. IRAS project ID:202216. All participants provided signed consent.

## Results

Three hundred and twenty-six couples with complete physical, social and behavioural characteristics and NMR data were included in the study. Characteristics of the participants are shown in Table 1. Couples exhibited correlations of varying strength for most physical, social and behavioural characteristics including age, height, alcohol consumption, education, smoking status, family history and ethnicity, with correlation coefficients ranging from 0.22 to 0.73 (Table 1). There was no evidence of within couple correlation for BMI and weight, where the correlation coefficients were − 0.03 (95% CI − 0.14, 0.08) and 0.01 (95% CI − 0.10, 0.12) respectively (Table 1). When we repeated the analyses after excluding couples (n = 17) where the male BMI was > 30 kg/m^2^, the results were unchanged with a correlation for BMI of − 0.02 (95% CI − 0.13, 0.09), p = 0.75.

**Table 1:** Demographic/lifestyle characteristics of couples undergoing IVF treatment (N = 326).

	Females	Males	Correlation coefficient (95% CI)	P value for association between traits in couples*
Age (years)	35.6 (4.4)	37.2 (5.7)	0.61 (0.53, 0.69)	< 0.001
Height (cm)	164.2 (6.3)	176.5 (5.1)	0.22 (0.12, 0.32)	< 0.001
Weight (kg)	66.7 (9.7)	78.6 (10.2)	0.01 (− 0.10, 0.12)	0.86
BMI (kg/m^2^)	24.7 (3.24)	25.2 (3.0)	− 0.03 (− 0.14, 0.08)	0.55
Alcohol (units per week)	4 (1,8)	4 (2,9)	0.62 (0.50, 0.74)	< 0.001
**Education**				
School	146 (45%)	147 (45%)		
Undergraduate	115 (35%)	139 (43%)		
Postgraduate	65 (20%)	40 (12%)		< 0.001
**Smoking**				
Ever	83 (25%)	97 (30%)		
Never	243 (75%)	229 (70%)	0.47 (0.36, 0.57)	< 0.001
**Physical activity (times per week)**				
Never/once	33 (10%)	29 (9%)		
Twice	70 (21%)	73 (22%)		
3–4 times	196 (60%)	177 (54%)		
> 4 times	27 (8%)	47 (14%)		< 0.001
**Family history of cardiometabolic disease**				
Yes	167 (51%)	154 (47%)		
No	159 (49%)	172 (53%)	0.39 (0.29, 0.50)	< 0.001
**Ethnicity**				
White	299 (92%)	300 (92%)		
Non-white	27 (8%)	26 (8%)	0.73 (0.59 ,0.88)	< 0.001

*From Pearson’s correlation/chi square test.

**Figure 2:**
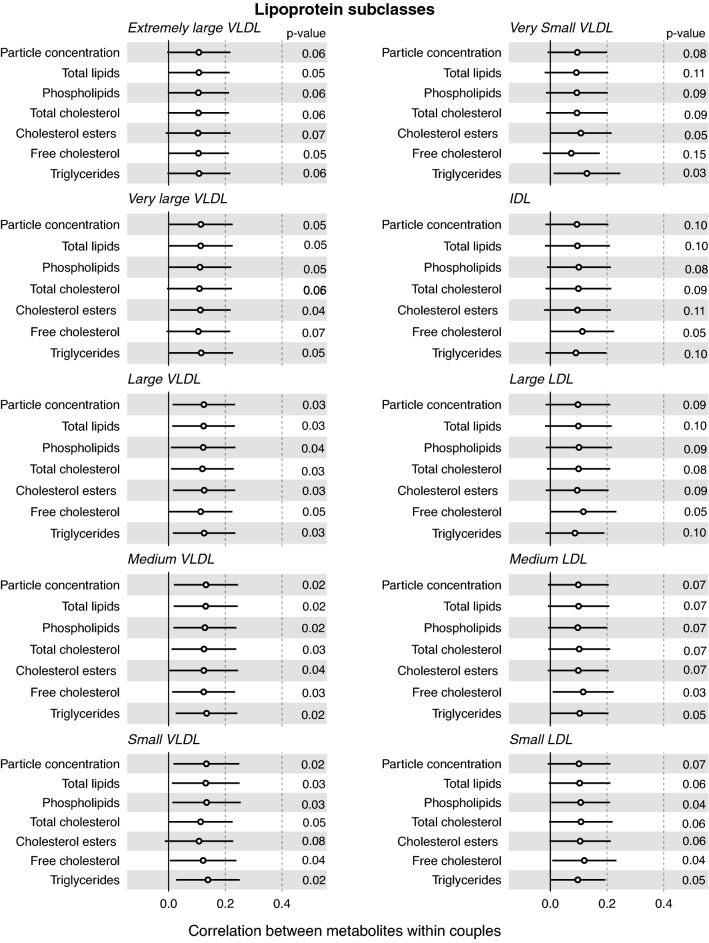

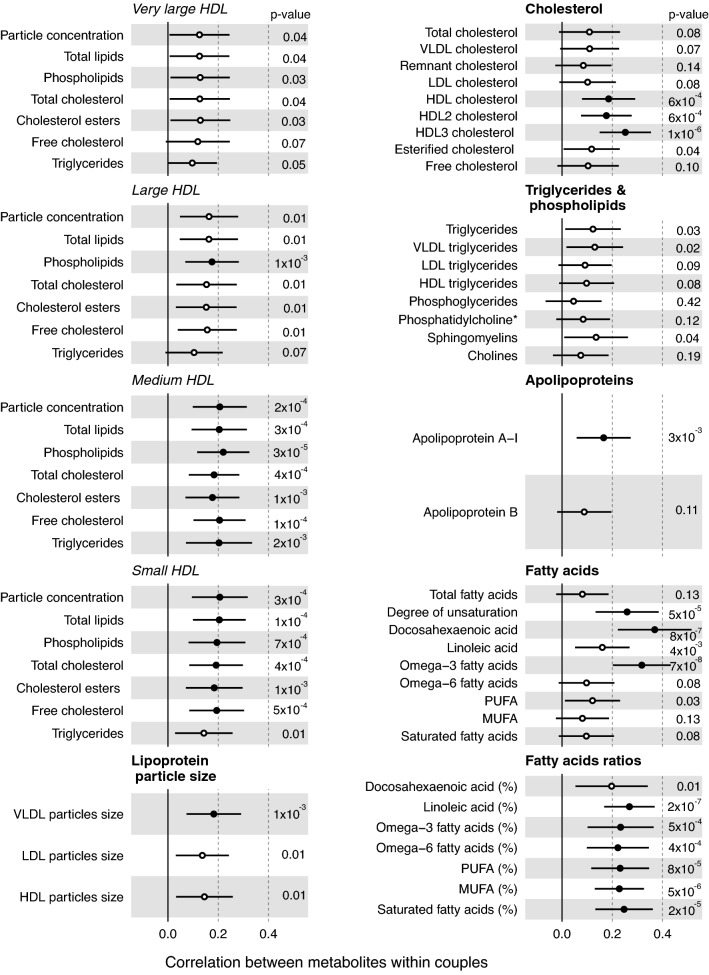

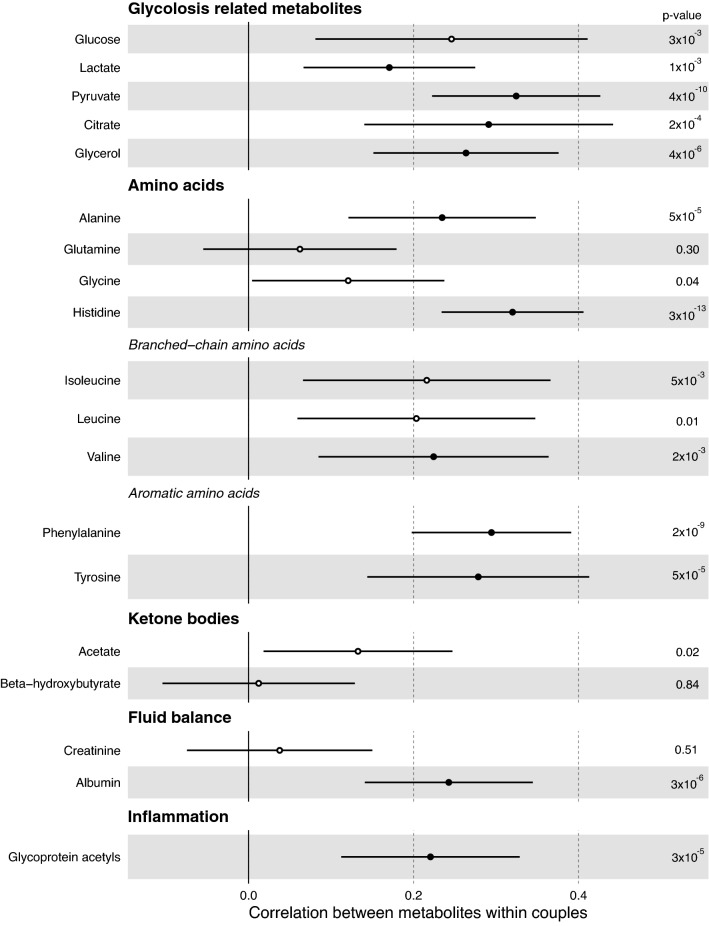
Correlations of lipoprotein classes, fatty acids and metabolic traits in couples awaiting IVF. Pearson correlation coefficients and 95% CI of within couple.

The correlation estimates for the unadjusted metabolite measures are shown in Fig. 2, with overall similar results for the Spearman correlation coefficients (Supplemental Fig. 1). Across the metabolites correlation point estimates were all positive and ranged from very weak to modest, with the median coefficient across all 155 measures being 0.12 (full range 0.01–0.37 and interquartile range 0.10–0.18). For lipoproteins, the correlation coefficients ranged from 0.11 for very large VLDL and 0.13 for medium VLDL, to 0.12–0.21 for very large HDL, large HDL, medium HDL and small HDL. For fatty acids the overall degree of unsaturation was correlated within couples (0.26, 95% CI 0.13, 0.38). Of the individual fatty acids docosahexaenoic acid (0.37, 95% CI 0.22, 0.52) and omega-3-fatty acids (0.32, 95% CI 0.20, 0.43) exhibited modest correlations within couples, with the correlation of docosahexaenoic acid the strongest correlation across all of the NMR measures. The contributions of individual fatty acid classes to total fatty acid concentrations was broadly similar within couples with coefficients ranging from 0.20 to 0.27. For all the glycolysis related metabolites, there was modest evidence of positive correlations within couples with the strongest effects observed for pyruvate (0.32, 95% CI 0.22, 0.43), citrate (0.29, 95% CI 0.14, 0.44) and glycerol (0.26, 95% CI 0.15, 0.38), with the correlation for glucose 0.25 (95% CI 0.08, 0.41). Of the amino acids only, histidine had evidence of modest correlation (0.32 95% CI 0.23, 0.41), with alanine, isoleucine, leucine, valine, phenylalanine, glycine and tyrosine exhibiting weaker positive correlations (0.12–0.29) within couples.

**Figure 3:**
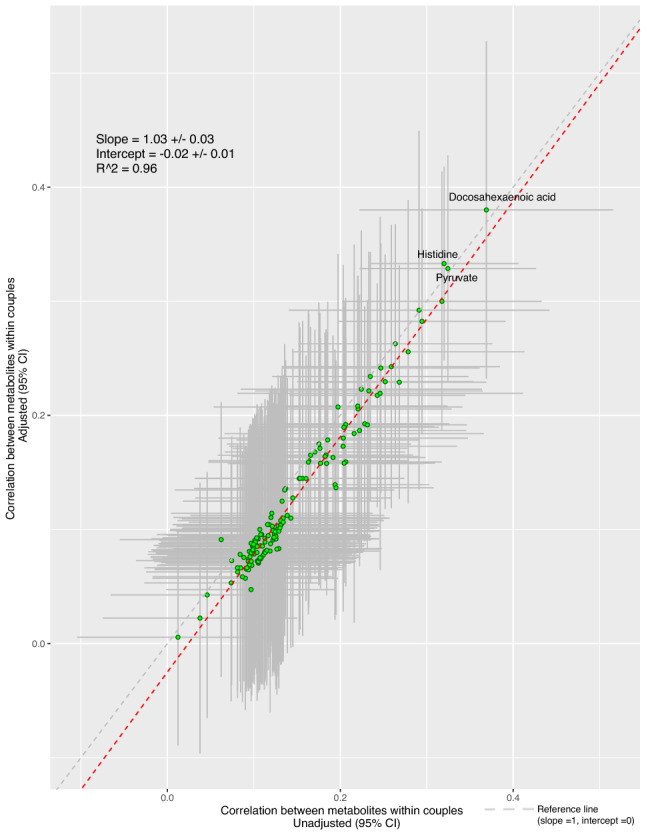
Scatterplot of adjusted versus unadjusted within couple correlation estimates across all metabolites. In adjusted analyses in males and females separately, metabolites (in SD units) were regressed on covariates (age, educational attainment, ever smoking, physical activity, family history of cardiometabolic disease, alcohol consumption, BMI, and ethnicity) and the residuals of those models use to estimate the adjusted Pearson correlation coefficients within couples. Unadjusted analyses were the Pearson correlation coefficients of the metabolites in SD units. The green dots highlight the individual metabolites. The grey dashed line reflects the reference line (slope 1, intercept 0) and the red dashed line is the best line of fit.

Correlation estimates within couples were similar in adjusted versus unadjusted analyses (Fig. 3) for most metabolites (R^2^ = 0.96 across all metabolites coefficients). For adjusted analyses the results were similar when covariates were adjusted by linear or quantile regression (Supplemental Figs. 2, 3 respectively).

**Figure 4:**
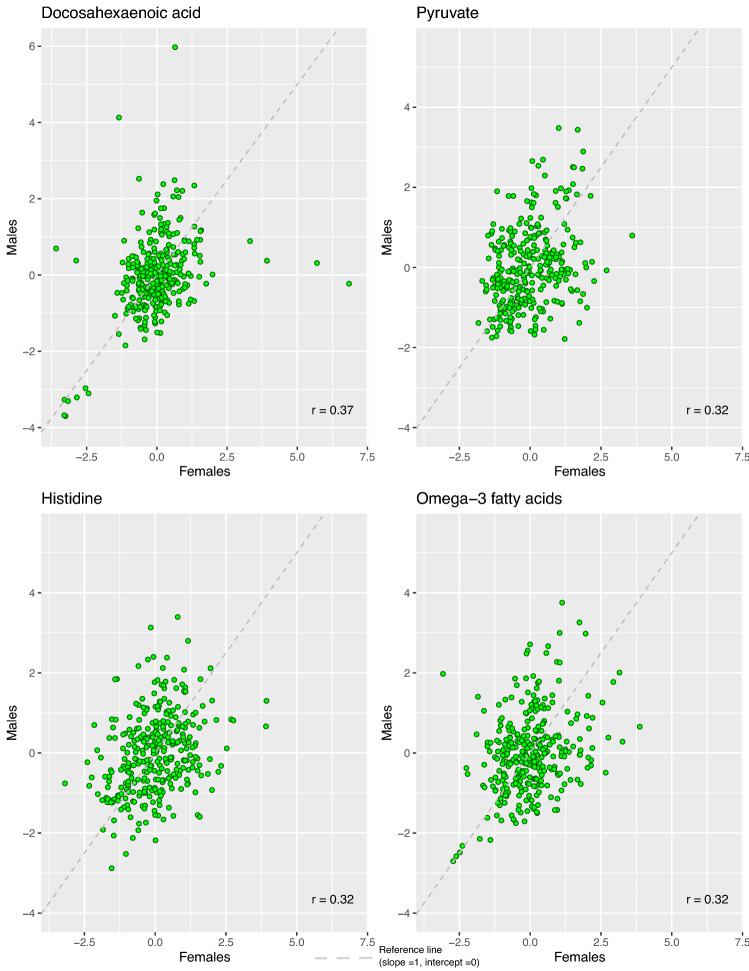
Multi-panel scatterplot of individual measures of metabolites in 326 women and their male partners for four selected metabolites with a within couple correlation of greater than 0.3. The green dots highlight the individual couples. The grey dashed line reflects the reference line (slope 1, intercept 0) and the red dashed line is the best line of fit.

Figure 4 shows the scatter plots of the unadjusted metabolite concentrations (in SD units) for each woman versus her male partner for the four metabolites with a Pearson’s correlation of greater than 0.3; Docosahexanoic acid, pyruvate, histidine and omega-3-fatty acids. For all four metabolites the concentrations were mostly concentrated around central values in both women and men but with a spread showing the weak to moderate correlations.

### Post-hoc analyses

When comparing Pearson with Spearman’s correlations we noticed that beta-hydroxybutyrate exhibited different correlation coefficients (Pearson = 0.01 (95% CI − 0.10, 0.13) versus Spearman = 0.26 (95% CI 0.16, 0.36)). On further investigation there was an obvious outlier for beta-hydroxybutyrate, with the difference attenuated by removal of the outlier: Spearman (adjusted) = 0.18, Pearson (adjusted) = 0.08. When males with a BMI > 30 kg/m^2^ were excluded, there was still no evidence of a correlation of BMI within couples (n = 309, rho = − 0.02 (− 0.13, 0.09), p = 0.75).

## Discussion

In this cross-sectional study we demonstrate that couples attending for infertility treatment exhibit strong correlations for a range of physical, social and behaviour characteristics and modest to weak correlations for a range of lipids and some other metabolic measures. The similarity in correlations for height, education and ethnicity, with those found in couples not seeking fertility treatment^2–4,7,10^, suggest that conventional assortative mating is similar in infertile couples as in the general population. That diet is the principal source for several of the metabolites; docosahexaenoic acid, histidine, phenylalanine and omega-3-fatty acids, would suggest that convergence due to a shared environment and active co-participation in daily activities including food consumption facilitates convergence of some metabolites.

The present analyses provide additional evidence of assortative mating for age^32–34^, height^3^ and educational levels^2,7^, with strong evidence of endogamy with respect to self-declared ethnicity^11^. Age is well established as showing the greatest level of couple similarity among all personal characteristics, with spousal age correlations typically ranging from 0.70 to 0.90^11,32–34^. The reasons for our slightly lower estimate (0.61 95% CI 0.53, 0.69) are unclear but may reflect recruitment of participants with known fertility issues, as both maternal and paternal age are known to be independently inversely associated with fecundity, however, the median age gap was similar to that observed in the general population^35^. The observed modest estimate for height is similar to previous meta-analyses with moderate assortative pairing for height across human populations (r = 0.23, 95% CI 0.21, 0.23)^3^. For ethnicity although endogamy remains the norm in Scotland, it has declined over recent years with similar declines observed in other Western countries^11,36^. IMost studies including the current report have indicated a sustained increase in educational homogamy^34^, with moderate partner similarities for potential drivers for education including socioeconomic status, abilities and intelligence all documented^11^. Despite previous meta-analyses suggesting weak associations (r = 0.10–0.15) for BMI, weight and related indices including waist circumference and waist to hip ratios^8^, we did not observe any correlation. This is likely to reflect our unique population, as restriction of analyses to couples where the male BMI was also ≤ 30 kg/m^2^ did not change our findings.

The observed convergence of additional lifestyle factors like alcohol consumption (r = 0.62, 95% CI 0.50, 0.74), with a dominance of consumption of a low number of units may in part reflect that the population were drawn from an infertility clinic where healthy preconceptual lifestyle behaviours may be anticipated. Meta-analyses have previously suggested an overall moderate similarity for alcohol use (r = 0.36)^37^, though levels of similarity observed in different studies have ranged from negligible to high. For exercise, studies have generally reported correlations between 0.15 and 0.30^38–40^ albeit some higher than 0.40^41,42^. That our observed correlation of smoking status (r = 0.47 95% CI 0.36, 0.57), is marginally higher than previous meta-analyses estimates r (r = 0.23, 95% CI 0.12, 0.36)^8^ may reflect our eligibility criteria, as in Scotland placement on the waiting list for public funding of fertility treatment is dependent on confirmation of non-smoking by cotinine breath testing for both partners.

Limited evidence from twin and family studies suggest that the heritability (h^2^; proportion of phenotypic variance due to genetic factors) of lipids and lipid-like molecules have a mean h^2^ levels of 47% (range from h^2^ = 0.11 to h^2^ 0.66), while for organic acids and derivatives the mean is 0.41 (0.14–0.72), essential amino acids 0.42 (0.23–06.4) and non-essential amino acids 0.39 (0.22–0.69)^19–22,43^. As direct genetic variation in metabolites profiles would not produce a correlation between couples due to the invisible nature of both genes and metabolites, our observed correlations are likely to be due to through indirect pathways including assortative mating for social and behavioural characteristics. In a systematic review for coronary risk factors, significant but low (upper limit of 95% confidence interval, maximal 0.10) spousal correlations were identified for total and LDL cholesterol and total triglycerides^8^. These meta-analysis estimates are very similar to ours; total cholesterol 0.07 vs 0.11, LDL cholesterol 0.06 vs 0.10 and triglycerides 0.08 vs 0.12, with the detailed NMR breakdown of the lipid subclasses and lipoproteins providing further similar estimates of spousal correlation for lipid metabolism. Inference on whether assortative mating and/or cohabitation and thereby a shared environment underlie these associations has been achieved by using marriage duration as a surrogate for a common environment and potential convergence^8^. These support initial indirect assortative mating (i.e. on social or behaviour factors that influence metabolism), and that a shared environment may further influence lipid metabolism but to a lesser degree^8^.

We observed weak to moderate spousal correlations for a range of essential amino acids and omega-3-fatty acids including the subtype docosahexaenoic acid all of which have diet as their principal source^44,45^. The sharing of a common household larder and most main meal is a potential mechanism by which couples have similarities in types of food, and nutrient intakes^46^. Although gender asymmetry in the spousal adoption of health-related dietary changes has been reported^47^, this may not apply to preconceptual diets where females may have a dominant role in preparation for pregnancy. Consistent with the suggestion that shared diet may have a critical influence, heritability variance estimates for circulating serum levels of histidine, docosohexaenoic acid, phenylalanine have been all lower than those observed for lipids, with environmental factors such as diet having a much greater contribution^43^.

Glucose, pyruvate, citrate, plus lactate and the glyceroneogenesis pathways were all weakly correlated. A meta-analyses of six studies, estimated that history of spousal diabetes was a risk factor for diabetes in their partner (effect estimate 1.26 (95% CI 1.08–1.45)^48^. A data mining study of 5,643 couples and 5643 non-couple pairs similarly found strong associations of having diabetes within couples (5.2% both of the couple had diabetes) than non-couples (0.1%)^49^. Heritability and shared environmental factors are proposed to account for at least half of the variability in normalised fasting glucose^50^, however, our study is unable to delineate their respective contributions to the weak association observed here.

Our studies adds to the small number^3^ of studies that have previously explored spousal metabolite correlations^19–21^. It has a similar sample size to one of those previous studies^19^ and examines a similar number of metabolic traits to two of them^19,21^. We do however acknowledge several limitations. Participants were couples awaiting IVF and this homogeneous relatively healthy population may have resulted in some selection bias and may mean that our results do not generalise to a general population of couples of reproductive age or same-sex populations. Replication of our findings in age-matched fertile couples, would elucidate whether the observed correlations are in part attributable to infertility. Previous population studies have suggested that regardless of sex composition of the partnership, all couples demonstrate substantial within couple similarity in demographics including for age, education, race/ethnicity, work hours, and earnings^51^. Determination of metabolite concentrations were undertaken on non-fasting samples taken in the morning. This was necessary to align with clinical processes for a population who are undergoing assisted conception, where caloric restraint may be detrimental. Replication of our findings with non-fasted samples would be useful, but comparison of fasting and postprandial samples of using the same NMR analysis platform have not differed notably, with on average, sex and fasting/postprandial states explaining 5.2% and 4.4% of the total variance, respectively^52^. Our analyses are cross-sectional and included couples within a narrow age range. With repeat assessments of couple correlations over time, or with cross-sectional data including couples with a wide age range and number of years of being together, it would be possible to explore the relative contributions of assortative mating and convergence on the weak metabolite correlations we have observed. Previous studies that have tried to explore this using marriage/cohabitation duration as a surrogate have found little evidence of any convergence for physical measures such as BMI or blood pressure, while behaviours such as smoking and alcohol converged during the initial period of a relationship prior to marriage/cohabitation, whereas convergence in physical activity was sustained throughout life^53,54^. The NMR platform used misses a proportion of the currently quantifiable metabolites in human serum/plasma, including markers of microbiota metabolism, vitamins, co-factors, and xenobiotics, that may be influenced by diet and preconceptual supplements. We do not have detailed dietary questionnaires, which would allow us to confirm our suggestion that a shared environment and common food would contribute to the observed correlations of metabolites.

We have explored within couple correlations of multiple metabolomic traits and find weak to modest positive correlations for the vast majority that are not influenced by adjustment for traits know to be influenced by assortative mating or shared couple behaviours. This suggests assortative mating, for example via genes linked to assortative characteristics such as height and education, might have some potential weak to modest impact on couples having similar metabolic traits. Whilst we acknowledge replication in a general population would be valuable the broadly similar within couple correlations of physical, social, and behavioural traits in these couples provides some evidence that our findings might be generalisable. Longitudinal studies would be valuable to fully explore the relative roles of assortative mating and convergence.

## Supplementary Information

Supplementary Information 1.

Supplementary Information 2.

## Data Availability

The datasets used and/or analysed during the current study are available from the corresponding author on reasonable request.
