# Supplementary material for: Spousal associations of serum metabolomic profiles by nuclear magnetic resonance spectroscopy
Source: Sci Rep. 2021 Nov 3;11:21587. doi: 10.1038/s41598-021-00531-z (PMC8566506; doi:10.1038/s41598-021-00531-z)
Supplement: Supplementary file 2 — Supplementary Information 2. [file 41598_2021_531_MOESM2_ESM.pdf]

SUPPLEMENTARY MATERIAL

Spousal associations of serum metabolomic profiles by nuclear magnetic resonance spectroscopy

Karema Al Rashid MBChB[1†], Neil Goulding PhD[2,3†], Amy Taylor PhD [2,3,4], Mary Ann Lumsden MD[1], Deborah A Lawlor PhD[2,3,4]*, Scott M Nelson PhD[1,4]*

[1] School of Medicine, University of Glasgow, UK G31 2ER

[2] MRC Integrative Epidemiology Unit at the University of Bristol, UK BS8 2BN

[3] Population Health Science, Bristol Medical School, UK

[4] NIHR Bristol Biomedical Research Centre, Bristol, UK

† Joint first authors

* Joint senior authors

Content	Page(s)	Description
**Tables**		
Table S1	3	List of NMR metabolomic measures assessed in this study, with their units of quantification
**Figures**		
Figure S1	10	Spearman correlations of metabolic measures in couples awaiting IVF
Figure S2	13	Adjusted correlations of metabolic measures in couples awaiting IVF using linear regression
Figure S3	16	Adjusted correlations of metabolic measures in couples awaiting IVF using quantile regression

Table S1: NMR metabolic measures

Molecular class	Lipid, lipoprotein or metabolite name	Units*
**Extremely large VLDL**	Concentration of chylomicrons and extremely large VLDL particles	mol/l
	Total lipids in chylomicrons and extremely large VLDL	mmol/l
	Phospholipids in chylomicrons and extremely large VLDL	mmol/l
	Total cholesterol in chylomicrons and extremely large VLDL	mmol/l
	Free cholesterol in chylomicrons and extremely large VLDL	mmol/l
	Triglycerides in chylomicrons and extremely large VLDL	mmol/l
**Very large VLDL**	Concentration of very large VLDL particles	mol/l
	Total lipids in very large VLDL	mmol/l
	Phospholipids in very large VLDL	mmol/l
	Total cholesterol in very large VLDL	mmol/l
	Cholesterol esters in very large VLDL	mmol/l
	Free cholesterol in very large VLDL	mmol/l
	Triglycerides in very large VLDL	mmol/l
**Large VLDL**	Concentration of large VLDL particles	mol/l
	Total lipids in large VLDL	mmol/l
	Phospholipids in large VLDL	mmol/l
	Total cholesterol in large VLDL	mmol/l l
	Cholesterol esters in large VLDL	mmol/l
	Free cholesterol in large VLDL	mmol/l
	Triglycerides in large VLDL	mmol/l
**Medium VLDL**	Concentration of large VLDL particles	mol/l
	Total lipids in small VLDL	mmol/l
	Phospholipids in small VLDL	mmol/l
	Total cholesterol in small VLDL	mmol/l
	Cholesterol esters in small VLDL	mmol/l
	Free cholesterol in small VLDL	mmol/l
	Triglycerides in small VLDL	mmol/l
**Small VLDL**	Concentration of very small VLDL particles	mol/l
	Total lipids in very small VLDL	mmol/l
	Phospholipids in very small VLDL	mmol/l
	Total cholesterol in very small VLDL	mmol/l
	Cholesterol esters in very small VLDL	mmol/l
	Free cholesterol in very small VLDL	mmol/l
	Triglycerides in very small VLDL	mmol/l
**IDl**	Concentration of IDL particles	mol/l
	Total lipids in IDL	mmol/l
	Phospholipids in IDL	mmol/l
	Total cholesterol in IDL	mmol/l
	Cholesterol esters in IDL	mmol/l
	Free cholesterol in IDL	mmol/l
	Triglycerides in IDL	mmol/l
**Large LDL**	Concentration of large LDL particles	mol/l

	Total lipids in large LDL	mmol/l
	Phospholipids in large LDL	mmol/l
	Total cholesterol in large LDL	mmol/l
	Cholesterol esters in large LDL	mmol/l
	Free cholesterol in large LDL	mmol/l
	Triglycerides in large LDL	mmol/l
**Medium LDL**	Concentration of medium LDL particles	mol/l
	Total lipids in medium LDL	mmol/l
	Phospholipids in medium LDL	mmol/l
	Total cholesterol in medium LDL	mmol/l
	Cholesterol esters in medium LDL	mmol/l
	Free cholesterol in medium LDL	mmol/l
	Triglycerides in medium LDL	mmol/l
**Small LDL**	Concentration of small LDL particles	mol/l
	Total lipids in small LDL	mmol/l
	Phospholipids in small LDL	mmol/l
	Total cholesterol in small LDL	mmol/l
	Cholesterol esters in small LDL	mmol/l
	Free cholesterol in small LDL	mmol/l
	Triglycerides in small LDL	mmol/l
**Very large HDL**	Concentration of very large HDL particles	mol/l
	Total lipids in very large HDL	mmol/l
	Phospholipids in very large HDL	mmol/l
	Total cholesterol in very large HDL	mmol/l
	Cholesterol esters in very large HDL	mmol/l
	Free cholesterol in very large HDL	mmol/l
	Triglycerides in very large HDL	mmol/l
**Large HDL**	Concentration of large HDL particles	mol/l
	Total lipids in large HDL	mmol/l
	Phospholipids in large HDL	mmol/l
	Total cholesterol in large HDL	mmol/l
	Cholesterol esters in large HDL	mmol/l
	Free cholesterol in large HDL	mmol/l
	Triglycerides in large HDL	mmol/l
**Medium HDL**	Concentration of medium HDL particles	mol/l
	Total lipids in medium HDL	mmol/l
	Phospholipids in medium HDL	mmol/l
	Total cholesterol in medium HDL	mmol/l
	Cholesterol esters in medium HDL	mmol/l
	Free cholesterol in medium HDL	mmol/l
	Triglycerides in medium HDL	mmol/l
**Small HDL**	Concentration of small HDL particles	mol/l
	Total lipids in small HDL	mmol/l
	Phospholipids in small HDL	mmol/l
	Total cholesterol in small HDL	mmol/l
	Cholesterol esters in small HDL	mmol/l
	Free cholesterol in small HDL	mmol/l

	Triglycerides in small HDL	mmol/l
**Lipoprotein particle size**	Mean diameter for VLDL particles	nm
	Mean diameter for LDL particles	nm
	Mean diameter for HDL particles	nm
**Cholesterol concentrations**	Total cholesterol	mmol/l
	Total cholesterol in VLDL	mmol/l
	Remnant cholesterol (non-HDL and non-LDL cholesterol)	mmol/l
	Total cholesterol in LDL	mmol/l
	Total cholesterol in HDL	mmol/l
	Total cholesterol in HDL2	mmol/l
	Total cholesterol in HDL3	mmol/l
	Esterified cholesterol	mmol/l
	Free cholesterol	mmol/l
**Glycerides and phospholipid concentrations (and one ratio)**	Total triglycerides	mmol/l
	Triglycerides in VLDL	mmol/l
	Triglycerides in LDL	mmol/l
	Triglycerides in HDL	mmol/l
	Total phosphoglycerides	mmol/l
	Ratio of triglycerides to phosphoglycerides	
	Phosphatydilcholine and other cholines	mmol/l
	Sphingomyelins	mmol/l
	Total cholines	mmol/l
**Apolipoprotein concentrations (and one ratio)**	Apolipoprotein A-1	g/l
	Apolipoprotein B	g/l
**Fatty acid concentrations**	Total fatty acids	mmol/l
	Estimated degree of saturation	
	22:6, docosahexaenoic acid	mmol/l
	18:2 linoleic acid	mmol/l
	Omega-3 fatty acids	mmol/l
	Omega-6 fatty acids	mmol/l
	Polyunsaturated fatty acids	mmol/l
	Monounsaturated fatty acids; 16:1, 18:1	mmol/l
	Saturated fatty acids	mmol/l
**Fatty acid ratios**	Ratio of 22:6, docosahexaenoic acid to total fatty acids	%
	Ratio of 18:2 linoleic acid to total fatty acids	%
	Ratio of omega-3 fatty acids to total fatty acids	%
	Ratio of omega-6 fatty acids to total fatty acids	%
	Ratio of polyunsaturated fatty acids to total fatty acids	%
	Ratio of monounsaturated fatty acids to total fatty acids	%
	Ratio of saturated fatty acids to total fatty acids	%
**Glycolysis related metabolite**	Glucose	mmol/l
	Lactate	mmol/l
	Pyruvate	mmol/l
	Citrate	mmol/l
	Glycerol	mmol/l
	Alanine	mmol/l

	Glutamine	mmol/l
**Amino acid concentrations**	Glycine	mmol/l
	Histidine	mmol/l
branched	Isoleucine	mmol/l
branched	Leucine	mmol/l
branched	Valine	mmol/l
aromatic	Phenylalanine	mmol/l
aromatic	Tyrosine	mmol/l
**Ketone body concentrations**	Acetate	mmol/l
	Acetoacetate	mmol/l
	3-hydroxybutyrate	mmol/l
**Fluid balance marker**	Albumin	mmol/l
	Creatinine	mmol/l
**Inflammation marker**	Glycoprotein acetyls, mainly a1-acid glycoprotein	mmol/l

* These are the units used throughout the paper for each of the metabolic measures, unless we state that we are presenting results in standard deviation (SD) units. Where we present results that are the mean (in control participants) at 16-weeks these are the units. Where we present change in metabolic marker (between 16- to 36-weeks) or difference in change of metabolic markers the units are those listed in the table above per one week of gestational age.

VLDL: very low density lipoprotein; LDL: low density lipoprotein; IDL: intermediate density lipoprotein; HDL: high density lipoprotein

Supplementary Figures

Supplemental Figure S1. Spearman correlations of metabolic measures in couples awaiting IVF.

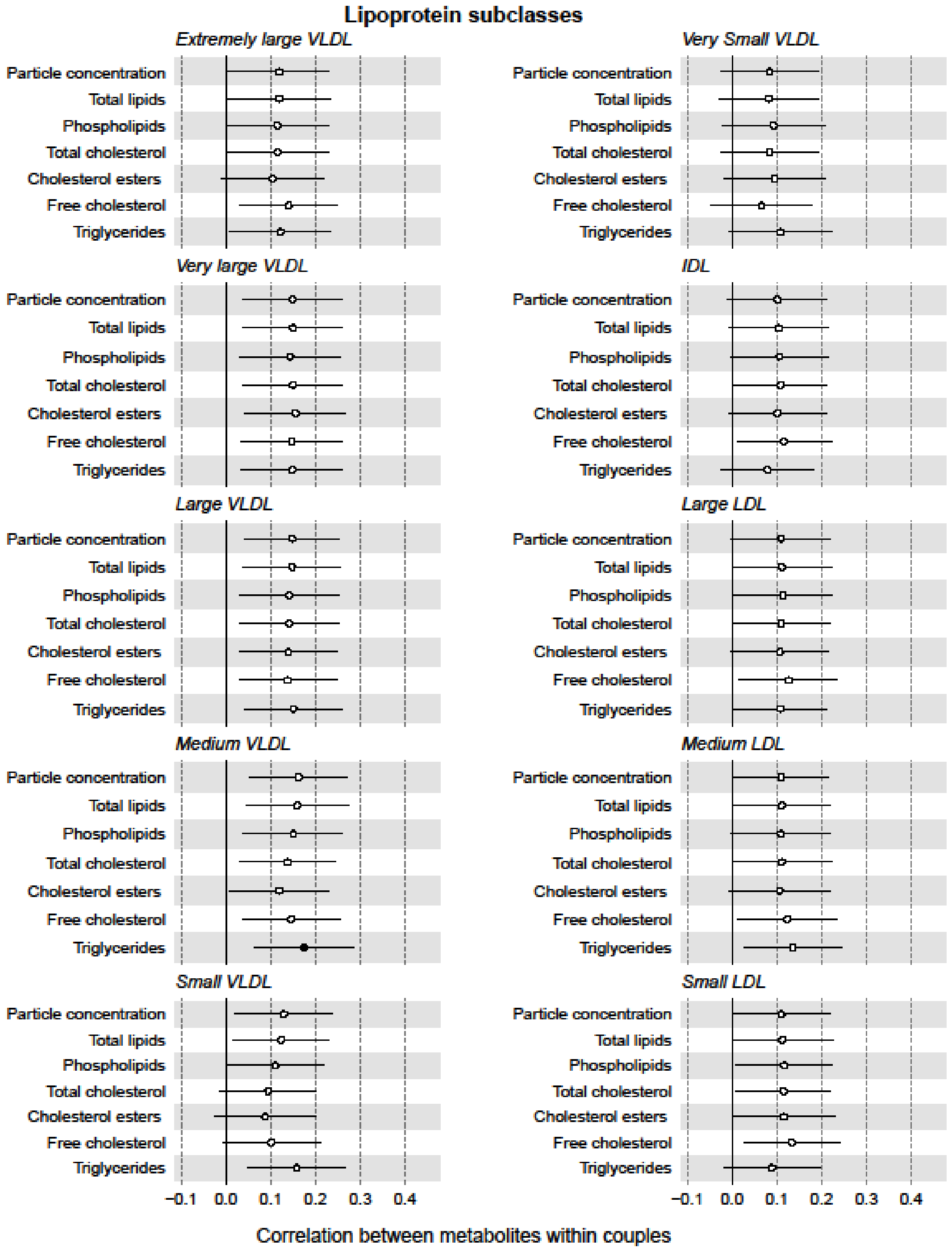

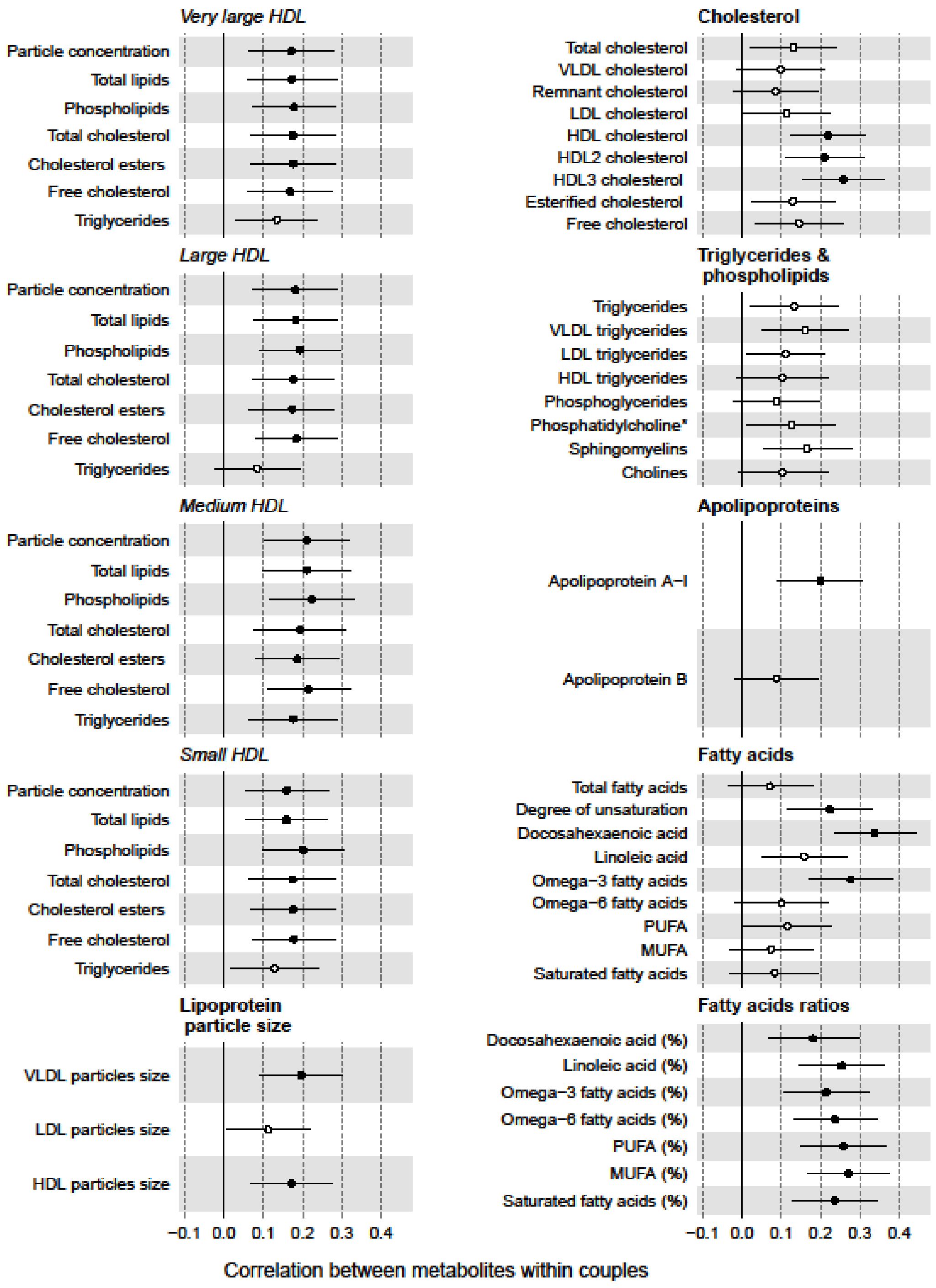
Very large HDL
Particle concentration
Total lipids
Phospholipids
Total cholesterol
Cholesterol esters
Free cholesterol
Triglycerides
Large HDL
Particle concentration
Total lipids
Phospholipids
Total cholesterol
Cholesterol esters
Free cholesterol
Triglycerides
Medium HDL
Particle concentration
Total lipids
Phospholipids
Total cholesterol
Cholesterol esters
Free cholesterol
Triglycerides
Small HDL
Particle concentration
Total lipids
Phospholipids
Total cholesterol
Cholesterol esters
Free cholesterol
Triglycerides
Lipoprotein particle size
VLDL particles size
LDL particles size
HDL particles size
Cholesterol
Total cholesterol
VLDL cholesterol
Remnant cholesterol
LDL cholesterol
HDL cholesterol
HDL2 cholesterol
HDL3 cholesterol
Esterified cholesterol
Free cholesterol
Triglycerides & phospholipids
Triglycerides
VLDL triglycerides
LDL triglycerides
HDL triglycerides
Phosphoglycerides
Phosphatidylcholine*
Sphingomyelins
Cholines
Apolipoproteins
Apolipoprotein A−I
Apolipoprotein B
Fatty acids
Total fatty acids
Degree of unsaturation
Docosahexaenoic acid
Linoleic acid
Omega−3 fatty acids
Omega−6 fatty acids
PUFA
MUFA
Saturated fatty acids
Fatty acids ratios
Docosahexaenoic acid (%)
Linoleic acid (%)
Omega−3 fatty acids (%)
Omega−6 fatty acids (%)
PUFA (%)
MUFA (%)
Saturated fatty acids (%)
−0.1 0.0 0.1 0.2 0.3 0.4
−0.1 0.0 0.1 0.2 0.3 0.4
Correlation between metabolites within couples

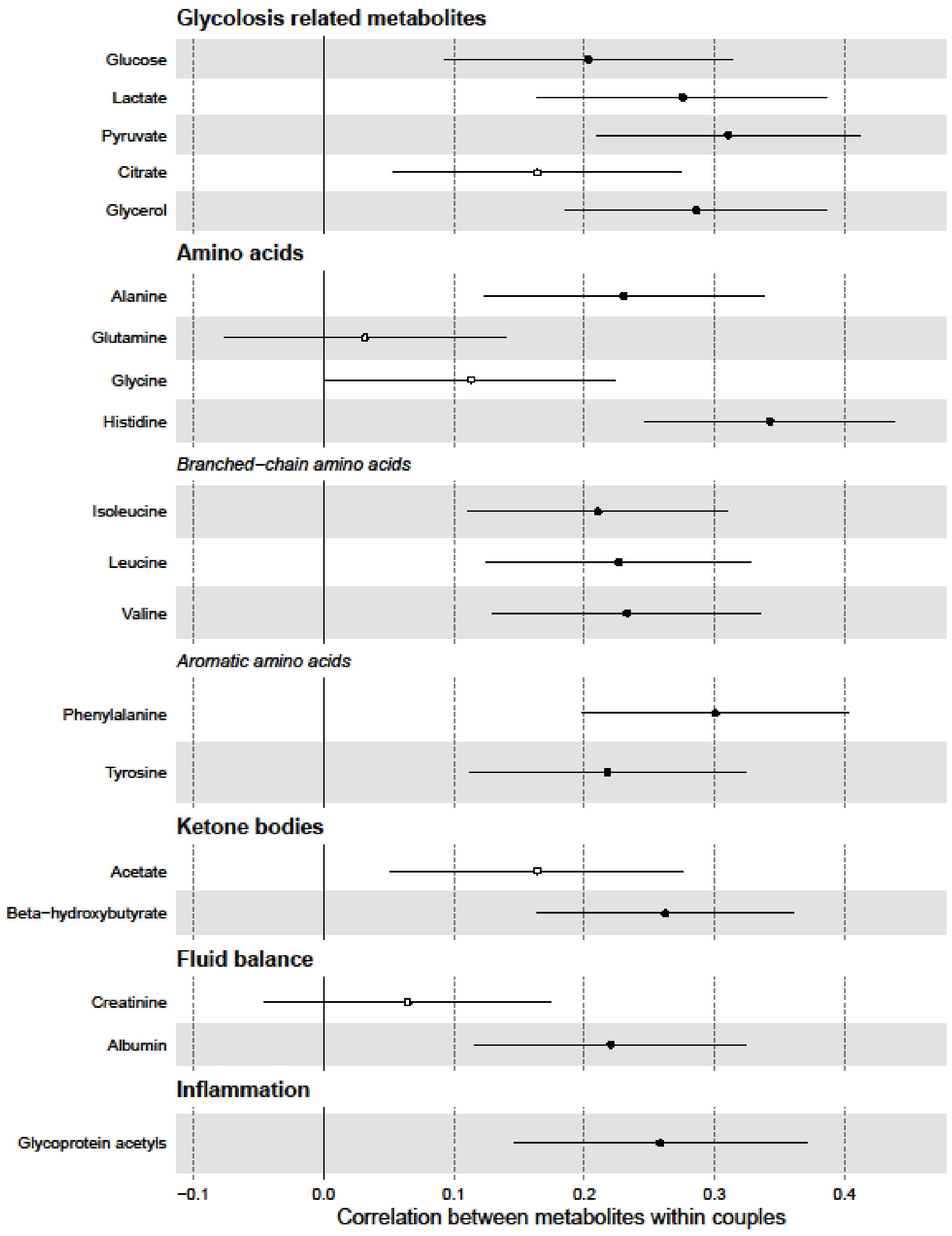

Glycolosis related metabolites
Glucose
Lactate
Pyruvate
Citrate
Glycerol
Amino acids
Alanine
Glutamine
Glycine
Histidine
Branched−chain amino acids
Isoleucine
Leucine
Valine
Aromatic amino acids
Phenylalanine
Tyrosine
Ketone bodies
Acetate
Beta−hydroxybutyrate
Fluid balance
Creatinine
Albumin
Inflammation
Glycoprotein acetyls
−0.1
0.0
0.1
0.2
0.3
0.4
Correlation between metabolites within couples

Supplemental Figure S2. Adjusted correlations of metabolic measures in couples awaiting IVF.

Pearson correlation coefficients and 95%CI of male and female linear regression residuals after sex-specific adjustment for age, educational attainment, ever smoking, physical activity, family history of cardiometabolic disease, alcohol consumption, BMI, and ethnicity.

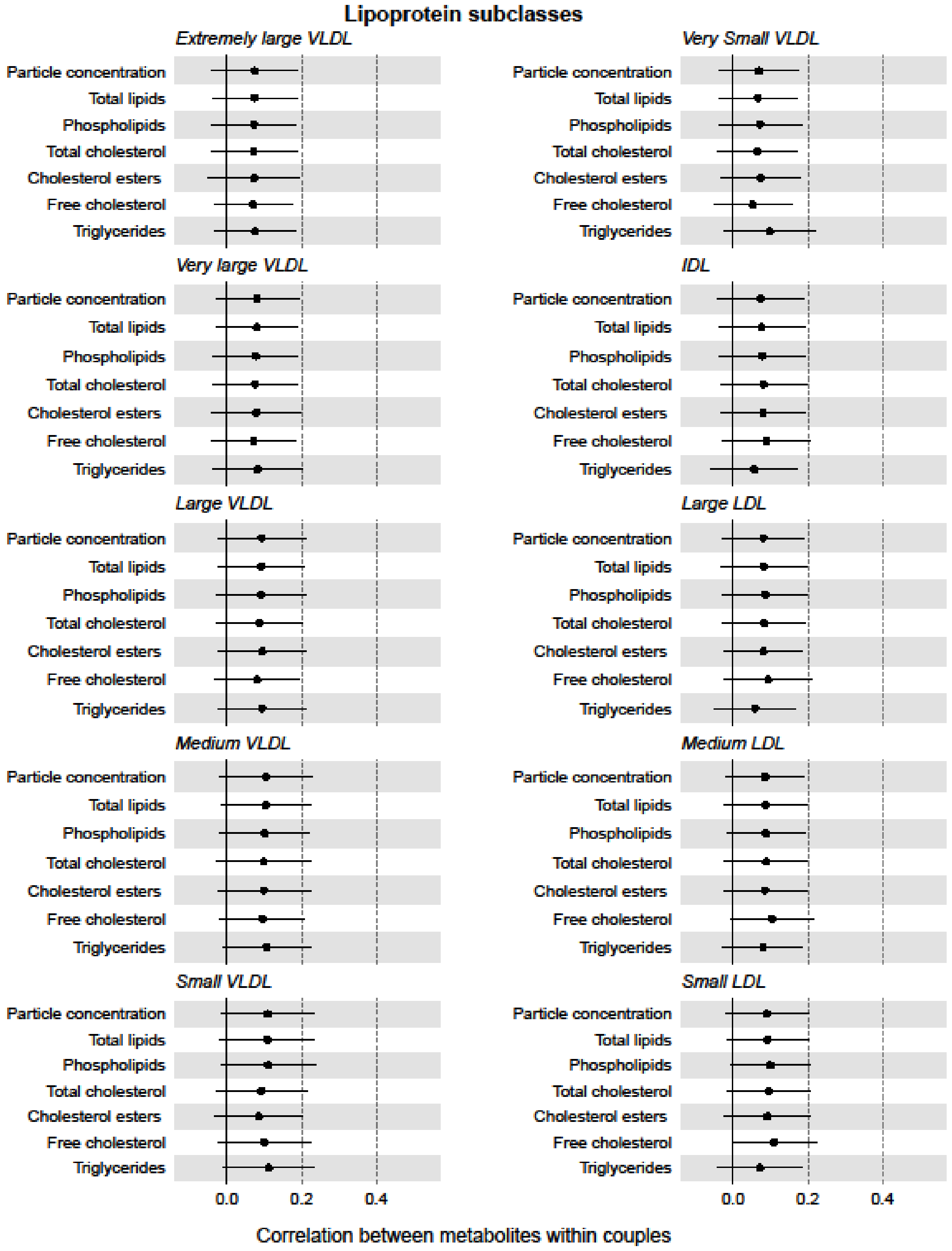

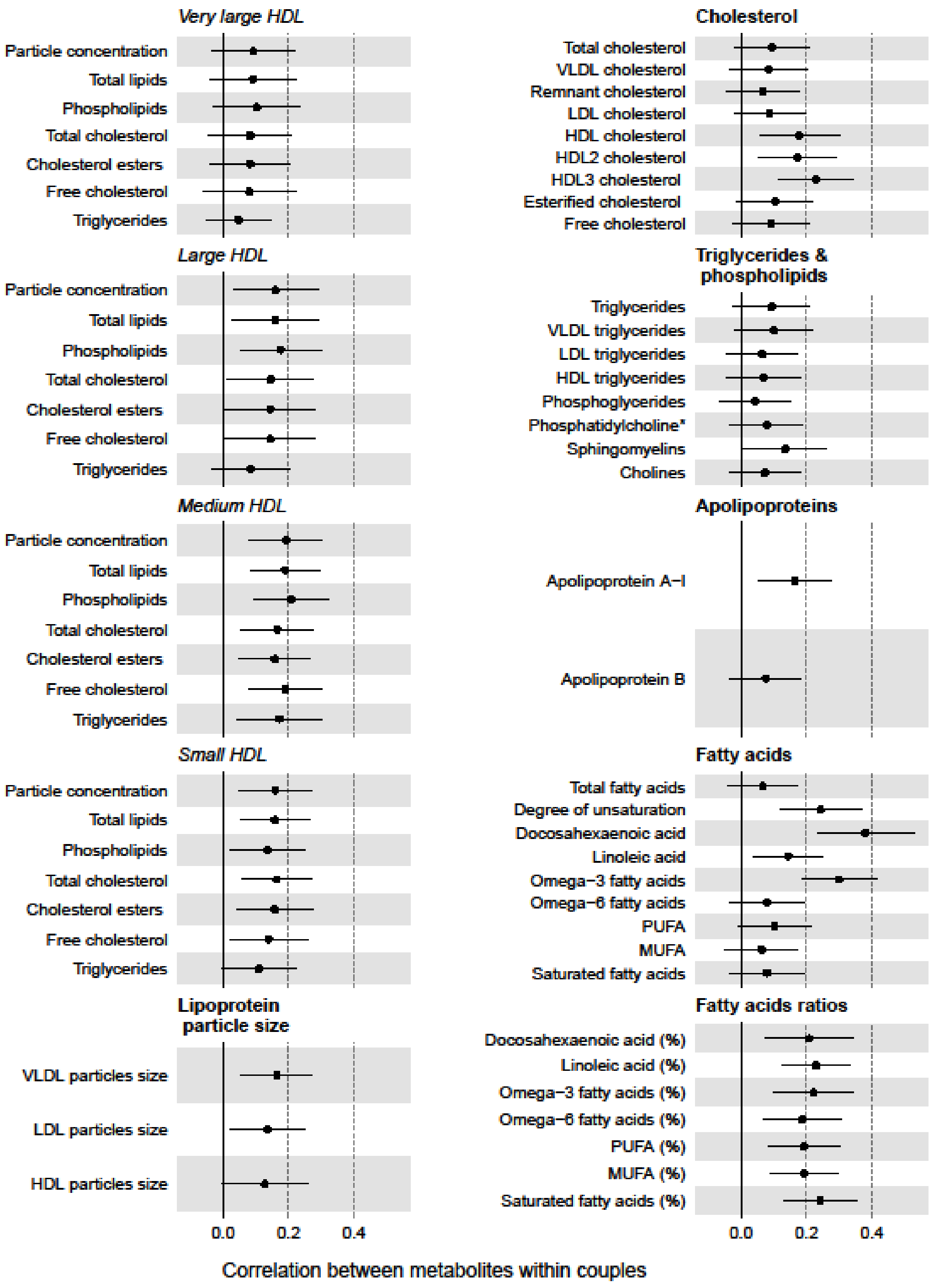
Very large HDL
Particle concentration
Total lipids
Phospholipids
Total cholesterol
Cholesterol esters
Free cholesterol
Triglycerides
Large HDL
Particle concentration
Total lipids
Phospholipids
Total cholesterol
Cholesterol esters
Free cholesterol
Triglycerides
Medium HDL
Particle concentration
Total lipids
Phospholipids
Total cholesterol
Cholesterol esters
Free cholesterol
Triglycerides
Small HDL
Particle concentration
Total lipids
Phospholipids
Total cholesterol
Cholesterol esters
Free cholesterol
Triglycerides
Lipoprotein particle size
VLDL particles size
LDL particles size
HDL particles size
Cholesterol
Total cholesterol
VLDL cholesterol
Remnant cholesterol
LDL cholesterol
HDL cholesterol
HDL2 cholesterol
HDL3 cholesterol
Esterified cholesterol
Free cholesterol
Triglycerides & phospholipids
Triglycerides
VLDL triglycerides
LDL triglycerides
HDL triglycerides
Phosphoglycerides
Phosphatidylcholine*
Sphingomyelins
Cholines
Apolipoproteins
Apolipoprotein A−I
Apolipoprotein B
Fatty acids
Total fatty acids
Degree of unsaturation
Docosahexaenoic acid
Linoleic acid
Omega−3 fatty acids
Omega−6 fatty acids
PUFA
MUFA
Saturated fatty acids
Fatty acids ratios
Docosahexaenoic acid (%)
Linoleic acid (%)
Omega−3 fatty acids (%)
Omega−6 fatty acids (%)
PUFA (%)
MUFA (%)
Saturated fatty acids (%)
0.0
0.2
0.4
0.0
0.2
0.4
Correlation between metabolites within couples

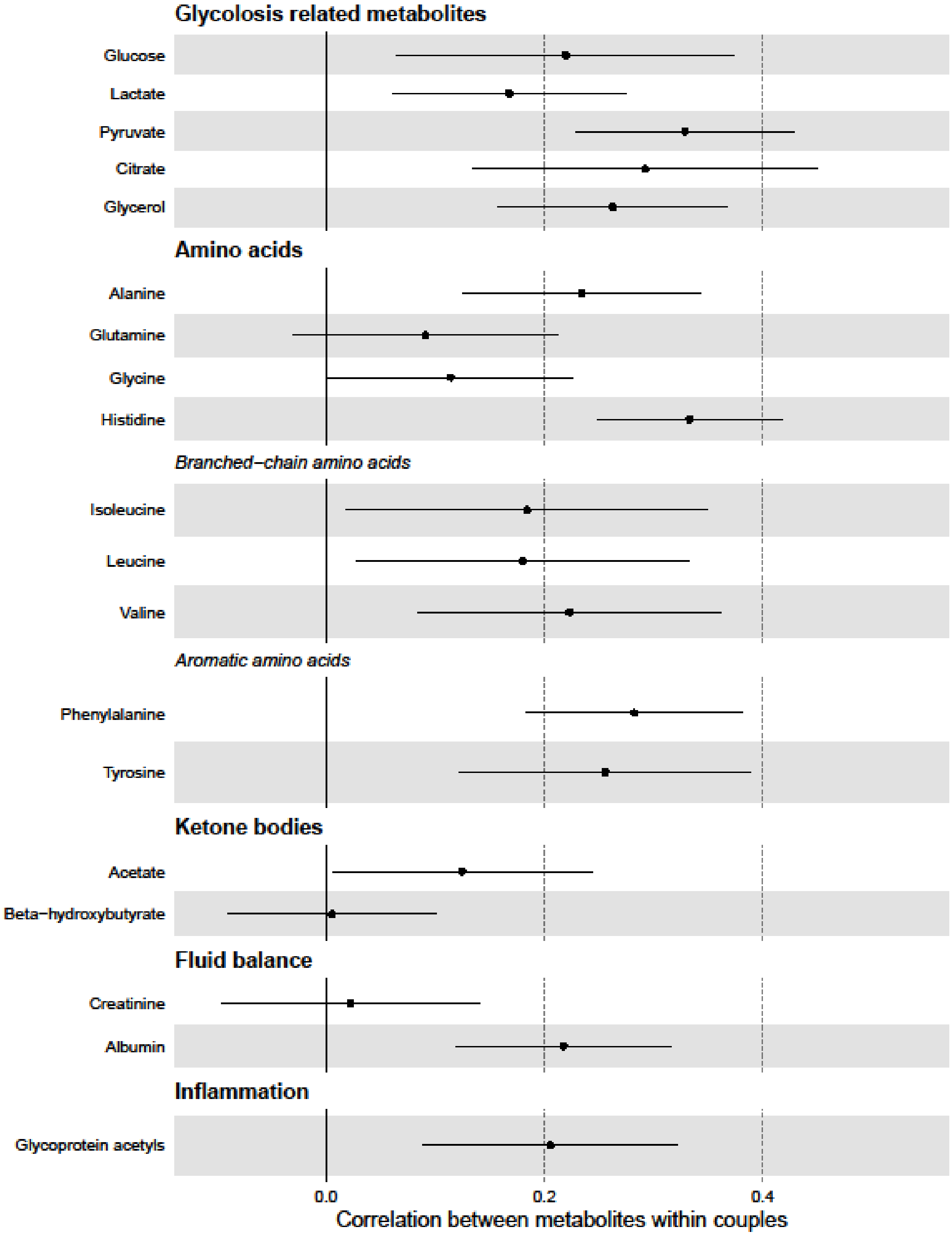

Glycolosis related metabolites
Glucose
Lactate
Pyruvate
Citrate
Glycerol
Amino acids
Alanine
Glutamine
Glycine
Histidine
Branched−chain amino acids
Isoleucine
Leucine
Valine
Aromatic amino acids
Phenylalanine
Tyrosine
Ketone bodies
Acetate
Beta−hydroxybutyrate
Fluid balance
Creatinine
Albumin
Inflammation
Glycoprotein acetyls
0.0
0.2
0.4
Correlation between metabolites within couples

Supplemental Figure S3. Correlations of metabolic measures in couples awaiting IVF using quantile regression.

Pearson correlation coefficients and 95%CI of male and female quantile regression residuals after sex-specific adjustment for age, educational attainment, ever smoking, physical activity, family history of cardiometabolic disease, alcohol consumption, BMI, and ethnicity.

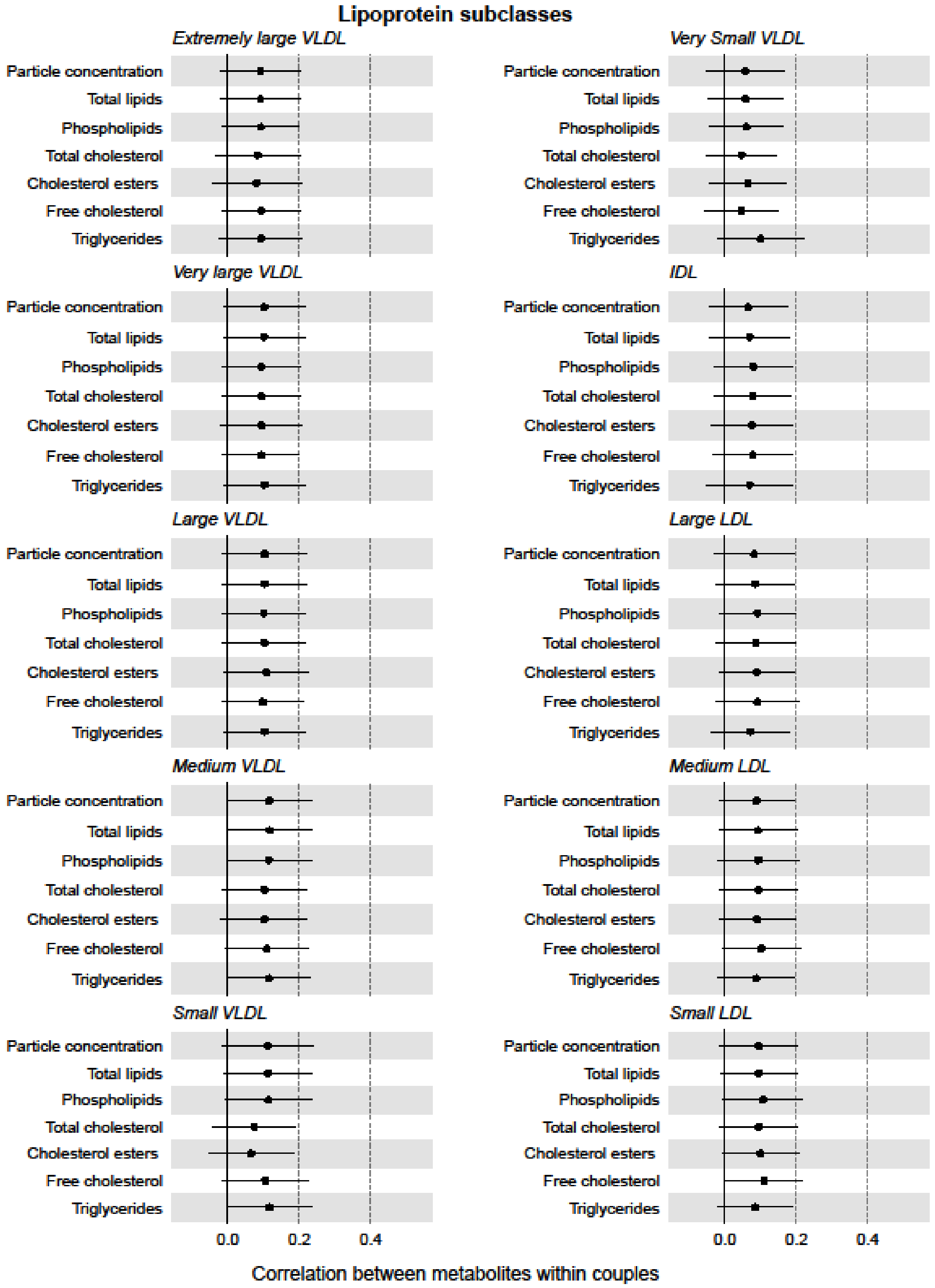

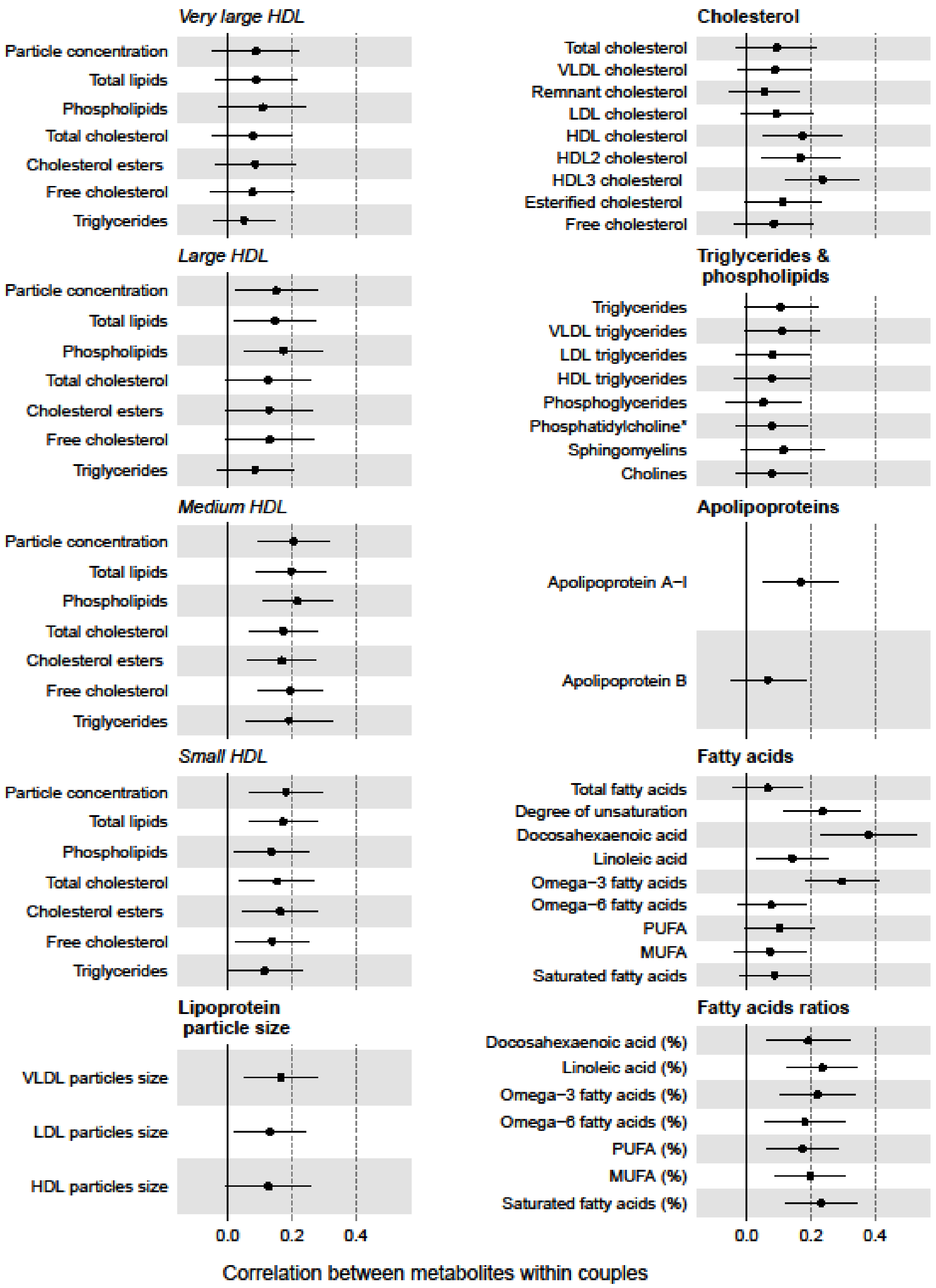
Very large HDL
Particle concentration
Total lipids
Phospholipids
Total cholesterol
Cholesterol esters
Free cholesterol
Triglycerides
Large HDL
Particle concentration
Total lipids
Phospholipids
Total cholesterol
Cholesterol esters
Free cholesterol
Triglycerides
Medium HDL
Particle concentration
Total lipids
Phospholipids
Total cholesterol
Cholesterol esters
Free cholesterol
Triglycerides
Small HDL
Particle concentration
Total lipids
Phospholipids
Total cholesterol
Cholesterol esters
Free cholesterol
Triglycerides
Lipoprotein particle size
VLDL particles size
LDL particles size
HDL particles size
Cholesterol
Total cholesterol
VLDL cholesterol
Remnant cholesterol
LDL cholesterol
HDL cholesterol
HDL2 cholesterol
HDL3 cholesterol
Esterified cholesterol
Free cholesterol
Triglycerides & phospholipids
Triglycerides
VLDL triglycerides
LDL triglycerides
HDL triglycerides
Phosphoglycerides
Phosphatidylcholine*
Sphingomyelins
Cholines
Apolipoproteins
Apolipoprotein A−I
Apolipoprotein B
Fatty acids
Total fatty acids
Degree of unsaturation
Docosahexaenoic acid
Linoleic acid
Omega−3 fatty acids
Omega−6 fatty acids
PUFA
MUFA
Saturated fatty acids
Fatty acids ratios
Docosahexaenoic acid (%)
Linoleic acid (%)
Omega−3 fatty acids (%)
Omega−6 fatty acids (%)
PUFA (%)
MUFA (%)
Saturated fatty acids (%)
0.0
0.2
0.4
0.0
0.2
0.4
Correlation between metabolites within couples

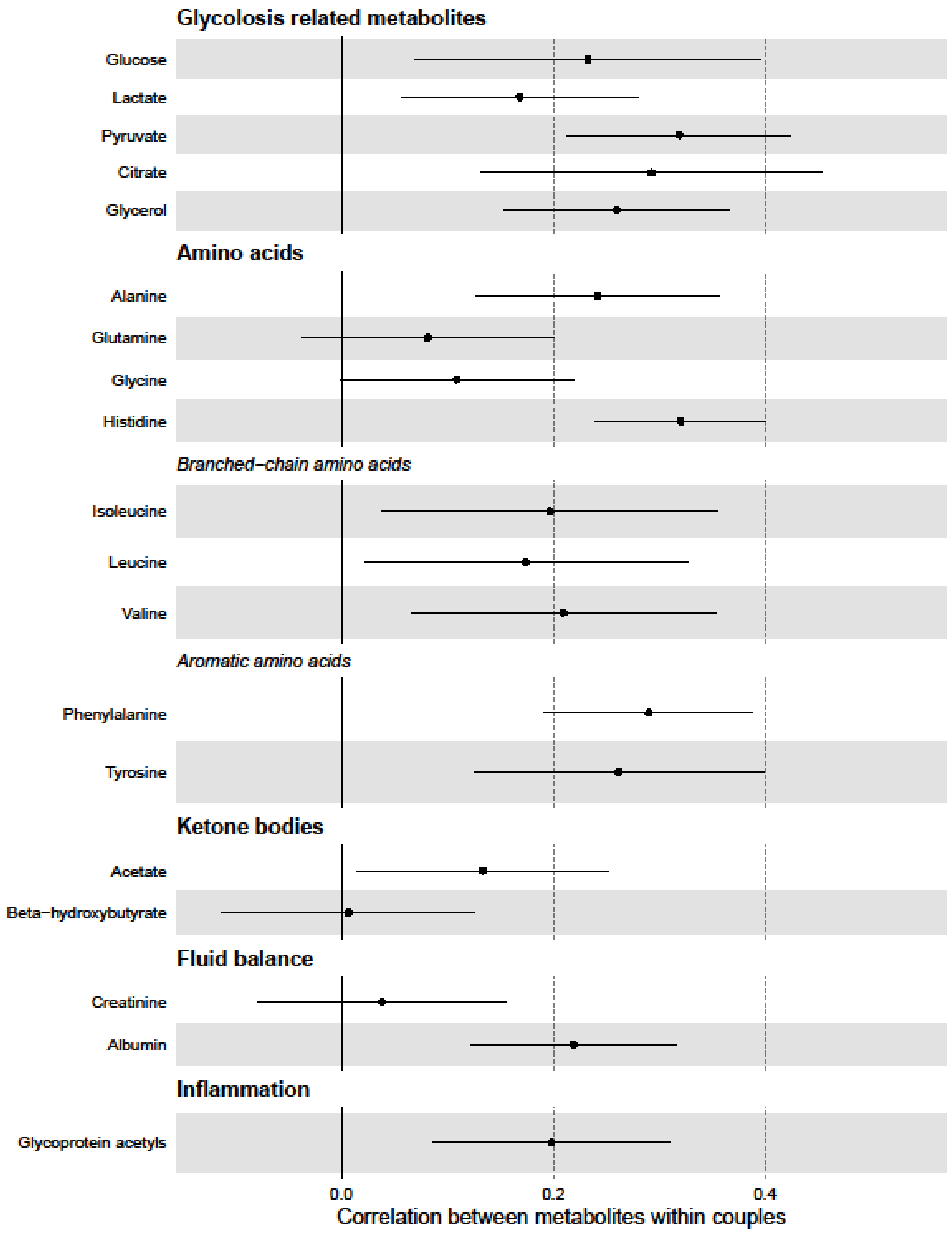
Glycolosis related metabolites
Glucose
Lactate
Pyruvate
Citrate
Glycerol
Amino acids
Alanine
Glutamine
Glycine
Histidine
Branched−chain amino acids
Isoleucine
Leucine
Valine
Aromatic amino acids
Phenylalanine
Tyrosine
Ketone bodies
Acetate
Beta−hydroxybutyrate
Fluid balance
Creatinine
Albumin
Inflammation
Glycoprotein acetyls
0.0
0.2
0.4
Correlation between metabolites within couples
